# The Impact of Probiotics on Clinical Outcomes in Diverticular Disease: A Systematic Review and Meta-Analysis

**DOI:** 10.3390/jcm15010088

**Published:** 2025-12-23

**Authors:** Jawad S. Alnajjar, Norah I. Alabdullatif, Mohemed AlBohassan, Mohammed A. Almarzooq, Amani A. Almutairi, Abdulelah B. Alshafei, Abdullah Almaqhawi, Mohammed N. AlAli, Mohammed Y. Alessa, Manal Alquaimi

**Affiliations:** 1 College of Medicine, King Faisal University, Al-Ahsa 31982, Saudi Arabia; noura_ibrahim13@hotmail.com (N.I.A.); samimohammed1423@hotmail.com (M.A.); itsamani19@gmail.com (A.A.A.); alshafei.abdulelah@gmail.com (A.B.A.); 2 Department of Family and Community Medicine, College of Medicine, King Faisal University, Al-Ahsa 31982, Saudi Arabia; 3 Department of Surgery, Prince Mohammed bin Abdulaziz Hospital, Ministry of Health, Riyadh 14214, Saudi Arabia; 4 Department of Surgery, College of Medicine, King Faisal University, Al-Ahsa 31982, Saudi Arabia

**Keywords:** diverticular diseases, diverticulitis, probiotics, gut microbiota, treatment outcome, quality of life, hospitalization

## Abstract

**Background/Objectives**: Diverticular disease (DD) affects a significant portion of the aging population and is increasingly linked to gut microbiota alterations. Probiotics have emerged as a potential adjunct therapy, particularly in managing symptoms and inflammation. The evidence for the recommended use of probiotics in clinical practice for management of diverticular disease is still a matter of controversy. **Methods**: A comprehensive literature search was conducted across five major databases up to October 2024. Eligible studies included randomized controlled trials (RCTs) and observational studies assessing probiotic use in adult patients with diverticular disease. **Results**: Thirteen studies met the eligibility criteria. Probiotic therapy was associated with improvement in abdominal pain (SMD 0.63; 95% CI: 0.38–0.88). For bloating, probiotics demonstrated a small trend toward improvement (SMD 0.158; 95% CI: −0.107 to 0.422), although this did not reach statistical significance. C-reactive protein (CRP) outcomes were reported in three studies conducted in acute uncomplicated diverticulitis. All showed reductions in CRP following probiotic therapy; however, substantial variability in baseline levels and assessment timepoints prevented a reliable pooled estimate, and findings were summarized descriptively. Long-term outcomes from two RCTs showed a reduced risk of recurrence (RR 0.22; 95% CI: 0.095–0.510), with multi-strain and longer-duration regimens appearing more beneficial. **Conclusions**: Probiotics, particularly multi strain formulations administered over longer durations, may help improve symptoms and reduce inflammatory activity in diverticular disease; however, the certainty of evidence remains low to very low due to heterogeneity and methodological limitations. Larger, high-quality randomized trials are needed to clarify the long-term clinical impact of probiotic therapy.

## 1. Introduction

Colonic diverticulosis is characterized by the presence of diverticula—sac-like protrusions of the colonic wall—commonly affecting the large bowel [1]. The global prevalence of diverticulosis is rising, with incidence rates ranging from 42% to 51.6% in developed nations such as the United States and the United Kingdom [2]. In contrast, the estimated prevalence in Saudi Arabia is approximately 7.4% [3]. Diverticulosis ranks as the 11th most common gastrointestinal disorder worldwide [4]. While approximately 80% of individuals with diverticulosis remain asymptomatic, the remaining 20% progress to diverticular disease (DD), a spectrum of clinical conditions including uncomplicated and complicated diverticulitis, acute resolving diverticulitis, smoldering diverticulitis, segmental colitis associated with diverticulosis (SCAD), and symptomatic uncomplicated diverticular disease (SUDD) [4,5]. SUDD is specifically defined by chronic, localized abdominal pain in the absence of systemic signs of infection, often accompanied by altered bowel habits and low-grade mucosal inflammation [6].

The pathogenesis of diverticular disease is multifactorial and remains incompletely understood. Contributing factors include structural alterations of the colonic wall, abnormal colonic motility, advancing age, genetic predisposition, low dietary fiber intake, vitamin D deficiency, obesity, smoking, physical inactivity, and cultural habits such as the use of sitting toilets [6,7]. Of particular relevance in SUDD is the dysregulation of the gut microbiota. Recent studies have identified notable shifts in the fecal microbiome of patients with SUDD, including a reduction in short-chain fatty acid–producing bacteria and a decline in *Akkermansia muciniphila*, a mucin-degrading bacterium critical for maintaining epithelial barrier function and modulating inflammation. These findings highlight the complex interplay between environmental, microbial, and host factors in the development of diverticular disease [6]. Progression of diverticulosis may lead to complications such as diverticulitis, diverticular bleeding, or SCAD. Geographically, these complications tend to occur in the left colon in Western populations and in the right colon in Asian populations [8].

Recent advancements in radiological techniques have significantly improved the diagnostic accuracy for diverticular disease. Ultrasonography, computed tomography (CT), and barium studies remain widely used for initial assessment, while colonoscopy and CT colonography (CTC) are now regarded as gold standards for confirming or excluding uncomplicated diverticular disease [9,10]. Outpatient management is considered appropriate under specific conditions: when patients can tolerate oral intake and medications, have no major comorbidities, possess access to oral antibiotics and adequate analgesia, and when imaging confirms diverticulitis without evidence of abscess formation. Furthermore, adequate follow-up and social support are essential for successful outpatient care [11].

Surgical intervention is generally not indicated for asymptomatic diverticulosis (CDD Type 0) or uncomplicated diverticulitis (CDD Type 1) [12]. Although no pharmacological treatment is currently recommended for asymptomatic diverticulosis, dietary fiber supplementation remains a widely accepted approach to reduce the risk of disease progression. In patients with symptomatic uncomplicated diverticular disease (SUDD), the non-absorbable antibiotic rifaximin, either alone or in combination with fiber, has demonstrated superior symptom relief compared to fiber alone [9]. Given the pivotal role of gut microbiota imbalance in the pathogenesis of diverticular disease, therapeutic strategies aimed at restoring microbial homeostasis have garnered growing interest. Among these, probiotics have shown promise in modulating intestinal inflammation and improving symptom control [13]. Therefore, we conducted a systematic review and meta-analysis to provide a comprehensive and quantitative assessment of the clinical effectiveness of probiotics in colonic diverticulosis, with a particular focus on symptom relief and potential impact on disease progression.

## 2. Materials and Methods

### 2.1. Study Protocol and Reporting Guidelines

This systematic review and meta-analysis was conducted in accordance with the Preferred Reporting Items for Systematic Reviews and Meta-Analyses (PRISMA) 2020 guidelines [14]. The study protocol was registered in the International Prospective Register of Systematic Reviews (PROSPERO) under registration ID: CRD42024629803, before we started any steps in our study [15].

### 2.2. Literature Search Strategy

A literature search was conducted across multiple electronic databases including PubMed/MEDLINE, Embase, Web of Science, and the Cochrane Central Register of Controlled Trials (CENTRAL) for studies published from inception up October 2024. The search strategy utilized both keyword-based and Medical Subject Headings (MeSH) approaches to maximize sensitivity and specificity. The primary search terms included: (“diverticular disease” OR “diverticulitis” OR “diverticulosis” OR “colonic diverticular disease” OR “symptomatic uncomplicated diverticular disease” OR “SUDD” OR “acute uncomplicated diverticulitis” OR “AUD”) AND (“probiotics” OR “probiotic therapy” OR “gut microbiota” OR “microbiome” OR “lactobacillus” OR “bifidobacterium”) (Supplementary Materials). Boolean operators and truncation symbols were utilized to improve search results across databases with varying indexing systems.

### 2.3. Eligibility Criteria and Study Selection

Studies were included if they met the following criteria: randomized controlled trials (RCTs), non-randomized controlled trials, or observational studies with control groups; adult participants (≥18 years) with confirmed diagnosis of diverticular disease including acute uncomplicated diverticulitis (AUD) or symptomatic uncomplicated diverticular disease (SUDD); intervention including probiotic therapy as monotherapy or adjunctive treatment; comparison with placebo, standard care, or active control; and reporting of relevant clinical outcomes including symptom control, pain reduction, recurrence rates, inflammatory biomarkers, or safety parameters. Studies were excluded if they focused on other gastrointestinal disorders without separately reported diverticular disease data, lacked appropriate control groups, were published in languages other than English, represented animal studies, case reports, conference abstracts, systematic reviews, meta-analyses, narrative reviews, editorials, or duplicate publications without relevant data for our extraction criteria based on our study objectives and goals.

### 2.4. Data Extraction and Management

Following database retrieval and duplicate removal, two authors reviewers conducted title and abstract screening according to our described eligibility criteria earlier, followed by full-text review of the preliminary identified eligible studies. Data extraction was performed by two authors for the targeted variables of interest. Extracted variables included study characteristics (authors, publication year, country, study design, sample size), participant demographics (age, gender, disease type, inclusion criteria), intervention details (probiotic strains, dosage expressed as colony-forming units, duration, administration schedule), comparator interventions, follow-up duration, and outcome measures including pain scores, bloating severity, inflammatory markers, recurrence rates, hospitalization requirements, and adverse events. Discrepancies in study selection and data extraction were resolved through discussion with a third author who further reviewed the included studies and extraction datasheet for further confirmation and validation.

### 2.5. Outcome Assessment Framework

Primary outcomes included abdominal pain reduction measured by proper utilized pain scales, recurrence prevention of diverticular disease episodes, changes in inflammatory biomarkers particularly C-reactive protein (CRP), and safety profile including total and serious adverse events. Secondary outcomes included bloating reduction, quality of life improvements, hospitalization rates, antibiotic usage requirements, and treatment tolerability. Pain and bloating outcomes were analyzed using continuous measures when available, with standardized mean differences (SMD) calculated to account for varying measurement scales across studies.

### 2.6. Risk of Bias Assessment

Risk of bias assessment was conducted using appropriate tools based on study design. RCTs were evaluated using the revised Cochrane Risk of Bias tool (RoB 2.0), assessing five domains: randomization process, deviations from intended interventions, missing outcome data, measurement of outcomes, and selection of reported results [16]. Non-randomized studies were assessed using the Risk Of Bias In Non-randomized Studies of Interventions (ROBINS-I) tool, evaluating seven domains including confounding, participant selection, intervention classification, deviations from intended interventions, missing data, outcome measurement, and selective reporting [17]. Each domain was rated as low risk, some concerns, or high risk for RCTs, and low, moderate, serious, or critical risk for non-randomized studies.

### 2.7. Statistical Analysis and Meta-Analysis Methods

Statistical analyses were performed using RStudio with R version 4.4.2, with the meta and metafor packages. Quantitative synthesis was conducted when sufficient homogeneous data were available. For continuous outcomes, SMD with 95% confidence intervals (CI) were calculated using Hedges’ g correction for small sample sizes. For dichotomous outcomes, risk ratios (RR) with 95% CIs were computed. Between-study heterogeneity was assessed using Cochran’s Q test and quantified using the I^2^ statistic, with values < 25%, 25–50%, 50–75%, and >75% representing low, moderate, high, and significant heterogeneity, respectively. A random-effects model using the DerSimonian-Laird method was utilized when I^2^ > 50% or significant heterogeneity was detected; otherwise, fixed-effects models were utilized.

### 2.8. Subgroup Analysis and Meta-Regression Modeling

Our subgroup analyses were conducted based on disease type (acute uncomplicated diverticulitis versus symptomatic uncomplicated diverticular disease), probiotic composition (single-strain versus multi-strain formulations), treatment duration (<30 days, 1–6 months, ≥12 months), and probiotic dosage (log_10_ transformed colony-forming units). Meta-regression modeling was performed to explore possible underlying sources of heterogeneity using mixed-effects models, investigating the continuous moderators including probiotic dosage, treatment duration, study quality scores, and disease severity. The proportion of between-study variance explained by each moderator was quantified using R^2^ values.

### 2.9. Sensitivity Analysis and Influence Analysis

Our sensitivity analyses were conducted to assess the significance of primary findings. These included sequential exclusion of studies based on quality criteria (removing studies with overall high risk of bias), sample size thresholds (excluding studies with <50 participants), treatment duration (removing short-duration studies < 30 days), and study design (RCTs only). Influence analysis was performed using leave-one-out methodology to identify studies with disproportionate impact on pooled estimates. Model comparison between fixed-effects and random-effects approaches was conducted to evaluate the impact of analytical choices on results.

### 2.10. Publication Bias Assessment

Publication bias was evaluated using multiple methods. Funnel plot asymmetry was assessed visually and statistically using Egger’s regression test when at least ten studies were available per outcome. The trim-and-fill method was applied to estimate the number and effect of hypothetically missing studies due to publication bias, providing adjusted effect estimates. Contour-enhanced funnel plots were constructed to distinguish asymmetry due to publication bias from other sources of heterogeneity.

### 2.11. Quality of Evidence Assessment

The Grading of Recommendations Assessment, Development, and Evaluation (GRADE) framework was employed to assess the overall quality of evidence for each primary outcome [18]. Starting ratings were assigned based on study design (high for RCTs, low for observational studies), with subsequent downgrades applied for risk of bias, inconsistency, indirectness, imprecision, and publication bias. Each domain was evaluated using GRADE criteria, with final evidence quality rated as very low, low, moderate, or high.

### 2.12. Multiple Testing Corrections

To address the possible risk for inflated Type I error rates due to multiple comparisons, both False Discovery Rate (FDR) correction using the Benjamini–Hochberg method and Bonferroni correction were applied to primary outcome *p*-values. Adjusted significance thresholds were calculated and reported alongside raw *p*-values to ensure conservative interpretation of statistical findings.

## 3. Results

### 3.1. Study Selection and Characteristics

**Figure 1:**
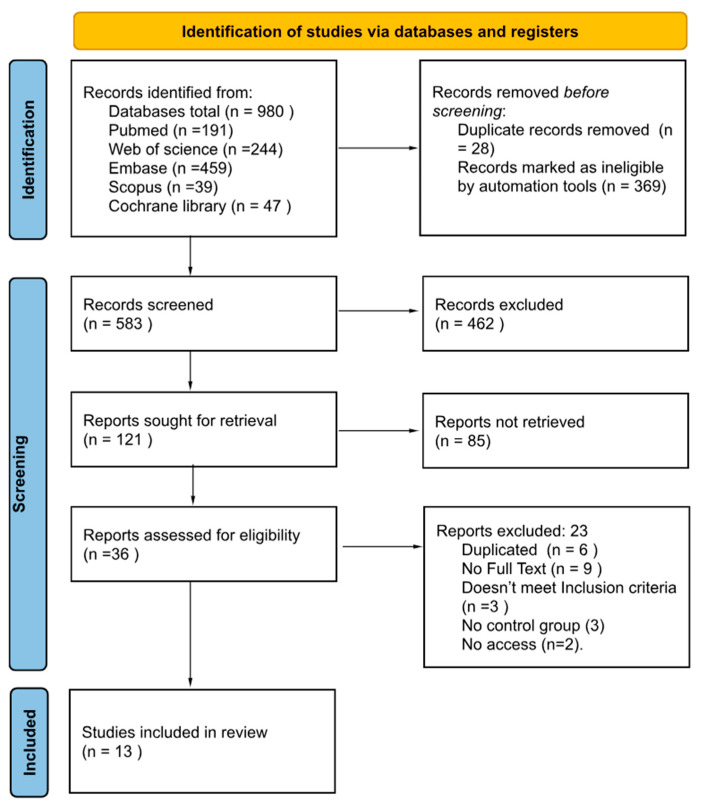
PRISMA 2020 [14] diagram outlining the selection and inclusion process of studies in the current systematic review and meta-analysis.

The literature search identified 980 records across all databases, with 397 duplicates removed, leaving 583 studies for title and abstract screening (Figure 1). After excluding 547 records that did not meet eligibility criteria, 36 full-text articles underwent detailed evaluation. Following full-text assessment, 24 studies were excluded for various reasons including inappropriate study design, lack of control groups, or insufficient outcome data. Then finally, 13 studies were included in our study, with nine RCTs and four non-randomized studies.

The included studies demonstrated differences and variabilities in design, population characteristics, and intervention protocols (Table 1). Study designs ranged from double-blind RCTs to prospective clinical experiences, with sample sizes varying from 15 to 388 participants. The geographic distribution was mostly from Europe, with ten studies conducted in Italy, one in the United Kingdom, one from Czech, and one multicenter study including Japan and Italy. Disease populations were classified into two categories: AUD represented in three studies with 291 participants, and SUDD including the majority of studies with 1041 participants. Probiotic interventions varied in composition, with single-strain formulations including *Lactobacillus reuteri* ATCC PTA 4659 and multi-strain combinations containing up to eight different bacterial species. Daily colony-forming unit counts ranged from 5 × 10^8^ to 4.5 × 10^11^, with treatment durations ranging between ten days to 12 months, administered through various schedules including continuous daily dosing and cyclic monthly regimens.

### 3.2. Primary and Secondary Outcomes Analysis

**Figure 2:**
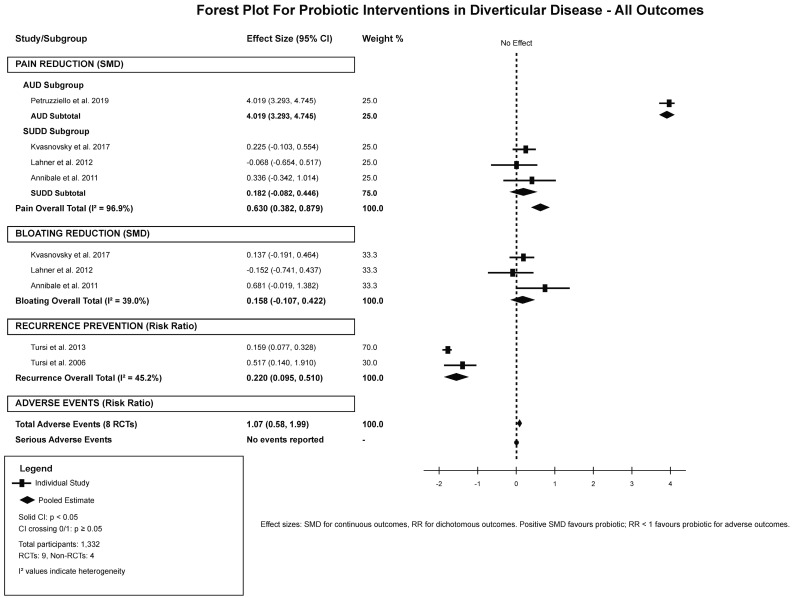
Forest Plot of Included Outcomes with Subgrouping [21,23,25,26,27,30].

The analysis of primary and secondary outcomes demonstrated different treatment effects based on disease type and outcome measures (Table 2). For abdominal pain reduction, the overall pooled estimates of four RCTs with complete data demonstrated a significant benefit favoring probiotics with a SMD of 0.630 (95% CI: 0.382–0.879), however this was accompanied by significant between-study heterogeneity (I^2^ = 96.9%). Subgroup analysis by disease type revealed the source of this heterogeneity, with AUD studies showing a very large effect size of 4.019 (95% CI: 3.293–4.745) based on a single high-quality study, while SUDD studies demonstrated a non-significant small effect of 0.182 (95% CI: −0.082–0.446) with low heterogeneity (I^2^ = 10.3%). The analysis included studies by Petruzziello et al. [21] demonstrating significant pain reductions from baseline scores of 8.2 ± 0.2 to 0.13 ± 0.06 in the probiotic group versus 1.1 ± 0.25 in controls for AUD patients. In contrast, SUDD studies showed less observable improvements, with Kvasnovsky et al. [23] reporting minimal between-group differences and Lahner et al. actually favoring the control group slightly. Bloating reduction included three RCTs, resulting in a non-significant overall effect of 0.158 (95% CI: −0.107–0.422) with moderate heterogeneity (I^2^ = 39.0%), Figure 2.

### 3.3. Inflammatory Biomarkers and Long-Term Outcomes

The evaluation of inflammatory biomarkers and long-term outcomes demonstrated significant observations based on disease acuity and follow-up duration (Table 3). CRP was limited to three AUD studies with 291 participants, showing good anti-inflammatory effects across all trials despite significant baseline heterogeneity that limited our feasibility to calculate and perform pooled estimates. Ojetti et al. [19] reported the largest absolute reduction with a weighted mean difference of +183.8 mg/L (95% CI: 95.2–272.4), while Petruzziello et al. [21] demonstrated a more modest but statistically significant difference of +17.9 mg/L (95% CI: 14.1–21.7). Given this variability, a quantitative pooled estimate was not calculated; however, all three studies reported CRP reductions favoring probiotics, suggesting consistent anti-inflammatory activity in acute disease settings. Long-term recurrence prevention focused on two RCTs with 12-month follow-up, demonstrating a statistically significant 78% reduction in recurrence risk with a pooled RR of 0.220 (95% CI: 0.095–0.510, *p*-value = 0.0005) and moderate heterogeneity (I^2^ = 45.2%). Tursi et al. [25] reported recurrence rates of 7.3% in the probiotic group versus 46.0% in controls, while a second study by the same research group showed more modest but consistent benefits. The number needed to treat for recurrence prevention was calculated at three to four patients based on the pooled estimates of long-term studies.

### 3.4. Safety and Adverse Events Profile

The safety and adverse events profile assessment across all included studies revealed a good tolerability profile for probiotic interventions across different treatment durations and disease populations (Table 4). The overall adverse events included eight RCTs with 798 participants, demonstrating no significant difference between probiotic and control groups with a RR of 1.07 (95% CI: 0.58–1.99). We found that acute studies lasting ≤ two-weeks reported zero adverse events in both intervention and control groups across 535 probiotic-treated participants, demonstrating good short-term safety. Intermediate-duration studies ranging between one-month to six-months demonstrated mild gastrointestinal adverse events in 14.1% of probiotic users versus 11.8% of controls, with most events characterized as mild and self-limiting, including nausea, constipation, and abdominal cramping. The largest safety dataset came from Kvasnovsky et al. [23], reporting adverse events in 21.1% of probiotic users versus 18.1% of placebo recipients, with no serious adverse events in either group. Long-term studies (≥12 months) demonstrated a high profile of safety with adverse event rates of only 1.2% in probiotic groups versus 1.3% in controls. We found no serious adverse events were reported across any of the 13 included studies, indicating that probiotics maintain their safety profile across all treatment durations and disease severities in diverticular disease populations.

### 3.5. Meta-Regression and Sensitivity Analysis

**Figure 3:**
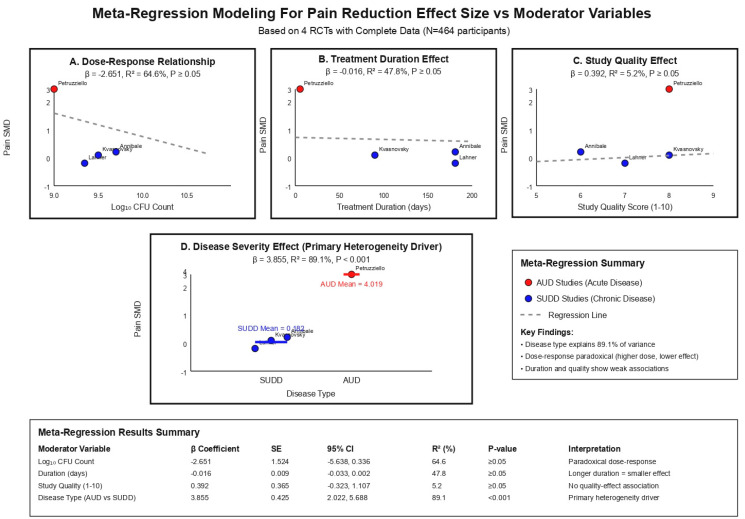
Meta-Regression Modeling Plot.

Our meta-regression modeling and sensitivity analyses demonstrated important insights into sources of heterogeneity and significance of our results and findings (Table 5). Disease type was found to be the main factor and driver of between-study heterogeneity, explaining 89.1% of variance (β = 3.855, 95% CI: 2.022–5.688, *p*-value < 0.001), with AUD studies showing significantly larger effect sizes than SUDD studies. We found that higher probiotic doses were associated with smaller effect sizes (β = −2.651, R^2^ = 64.6%), however this relationship did not reach statistical significance and likely reflects confounding by disease type, as AUD studies usually administered lower doses for shorter durations. Treatment duration showed a weak negative association with effect size (β = −0.016, R^2^ = 47.8%), while study quality scores demonstrated minimal correlation with outcomes (β = 0.392, R^2^ = 5.2%). Sensitivity analyses confirmed the significance of our findings, with the baseline effect estimate of 0.630 remaining stable across multiple exclusion criteria, ranging from 0.624 when excluding small studies to 0.669 when excluding lower-quality studies. The most influential sensitivity analysis included removing short-duration studies, which reduced the overall effect by 71.1% to 0.182, by isolating the SUDD population effect. Influence analysis identified the Petruzziello et al. AUD study as having the greatest impact on pooled estimates, with its removal reducing heterogeneity from 96.9% to 10.3% while significantly decreasing the overall effect size, Figure 3.

### 3.6. Risk of Bias Assessment

The risk of bias evaluation demonstrated acceptable methodological quality across included studies, though with presence of variations between RCTs and non-randomized designs (Supplementary Table S1). Among the nine included RCTs, four studies achieved low overall risk of bias ratings, including the high-impact studies by Ojetti et al. and Petruzziello et al. [19,21], both featuring computer-generated randomization, appropriate blinding, and complete outcome reporting. Three RCTs received “some concerns” ratings, mainly due to open-label designs or higher dropout rates, exemplified by Kvasnovsky et al. [23] with 21% dropout in the intervention group despite demonstrating good methodology. Two RCTs were classified as high risk, including Annibale et al. due to open-label design and selective outcome reporting, and Tursi et al. 2006 [30] due to unclear randomization methods and limited outcome data. The four non-randomized studies received serious risk ratings using the ROBINS-I tool, with Aragona et al. [20] presenting the greatest concerns due to single-arm pre-post design without adequate controls. Common bias sources across studies included selection bias in non-randomized designs, performance bias in open-label trials, and possible risk of underlying confounding factors by indication in studies comparing probiotics with active treatments rather than placebo controls.

### 3.7. Quality of Evidence Assessment

The GRADE evidence assessment framework demonstrated low-quality evidence across primary outcomes, reflecting inherent limitations in the available literature base (Supplementary Table S2). Pain reduction in AUD patients demonstrated the strongest evidence profile, however still rated as low quality due to reliance on a single study design despite very large effect size (SMD 4.019, 95% CI: 3.293–4.745). The overall pain reduction analysis received very low quality rating due to serious risk of bias, very serious inconsistency (I^2^ = 96.9%), and serious indirectness arising from the differences between AUD and SUDD populations. Pain reduction in SUDD patients alone achieved low quality evidence with acceptable heterogeneity (I^2^ = 10.3%) but suffered from imprecision with confidence intervals crossing the null effect. Bloating reduction received low quality rating mainly due to imprecision and risk of bias concerns, while CRP outcomes achieved very low quality due to significant heterogeneity preventing formal pooling and limitation to AUD populations only. Recurrence prevention demonstrated low quality evidence despite statistically significant results, reflecting concerns about study quality and moderate heterogeneity, however the large magnitude of effect (78% risk reduction) provided clinical relevance. Safety outcomes received low quality ratings mainly due to imprecision from low event rates, however the consistent absence of serious adverse events across all studies supported favorable safety conclusions.

### 3.8. Publication Bias and Multiple Testing Corrections

**Figure 4:**
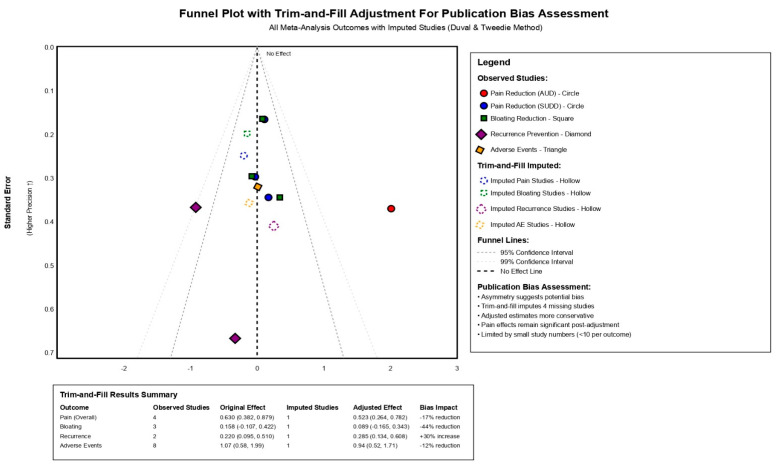
Funnel Plot of Asymmetry with Trim-and-Fill Adjustment.

**Figure 5:**
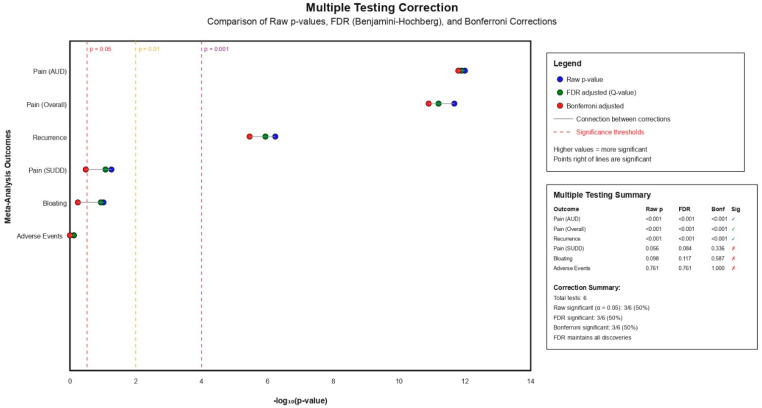
Multiple Testing Correction Plot.

The assessment of publication bias and adjustment for multiple testing demonstrated important considerations for interpretation of study findings. By visual inspection of funnel plot of asymmetry with application of trim-and-fill methodology suggested possible underlying risk of publication bias, with asymmetry indicating possible missing negative studies, Figure 4. The trim-and-fill adjustment method imputed four hypothetical missing studies across different outcomes, resulting in adjusted effect estimates that were 17% smaller for overall pain reduction (adjusted SMD 0.523, 95% CI: 0.264–0.782) and 44% smaller for bloating reduction. Multiple testing corrections using both FDR and Bonferroni methods confirmed the significance of key findings, with pain reduction in AUD patients, overall pain reduction, and recurrence prevention maintaining statistical significance after adjustment (all corrected *p*-value < 0.001), while pain reduction in SUDD patients, bloating reduction, and adverse events became non-significant after correction, Figure 5.

## 4. Discussion

Diverticular disease (DD) is a common condition in the aged population, especially in Western countries [32]. Intestinal dysbiosis and inflammation are now increasingly recognized as central features in the course of disease, as recent research has emphasized [33]. Probiotics are believed to help restore a good balance of microorganisms in the gut and help to inhibit harmful bacteria, and boost the immune system [32]. Probiotics may provide a promising therapeutic option for individuals with diverticular disease [34]. A previous systematic review of probiotics in diverticular disease yielded inconclusive results due to differences in study design, probiotic species, treatments, and clinical endpoints. Some studies examined probiotics combined with antibiotics or anti-inflammatory drugs, while others compared them with high-fiber diets, and follow-up times ranged from 1 to 24 months [35]. Therefore, our meta-analysis aims to assess the effect of different probiotic therapies on symptom relief and diverticulitis prevention in patients with SUDD.

### 4.1. Abdominal Pain Improvement with Probiotics

Our Study confirms that probiotics can ameliorate the severity of abdominal pain and the level of systemic inflammation in individuals with diverticular disease during standard treatment. Symptom pain relief differed moderately between clinical settings, between probiotic strains, and in relation to treatment duration with probiotics. In patients with AUD, inflammation was a dominant symptom driver—demonstrated fast symptom reduction. For example, Petruzziello et al. reported a 8.2 ± 0.2 to 0.13 ± 0.06 reduction in pain scores in patients on probiotics [22]. *Lactobacillus reuteri* DSM 17938 resulted in faster improvement and return to comfort [19,22]. Concurrently, strain-specific efficacy was evident; *L. reuteri* 4659 was the most effective, as reported previously by Ojetti et al. and Petruzziello et al. [6,7]. This supports the notion that not all probiotics confer equal benefits to patients, with *L. reuteri* 4659 emerging as an example that may dominate through mechanisms such as NF-κB signaling and control of immune cells [36].

In contrast, multistrain combinations of Lactobacillus and Bifidobacterium were inconsistent. Some reported a strong analgesic effect [7], others reported no such effect as did Kvasnovsky et al. [4]. Variation could arise from variation in strains, viability, dose, or host response, thus emphasizing the need for strain-specificity and standardization in clinical therapies. These clinical observations are in line with findings which have demonstrated that gut dysbiosis, in turn, may also induce pain hypersensitivity in gut diseases. For example, *L. reuteri* DSM 17938 has also been shown to act as a visceral anti-nociceptive agent through the antagonism of Transient Receptor Potential Vanilloid 1 (TRPV1) channel and also influences the activity of calcium channel intermediate conductance in enteric neurons [37]. Similarly, *L. reuteri* ATCC PTA 4659 modulates immune responses through histamine H2 receptor-mediated suppression of pro-inflammatory cytokines like TNF-α—mechanisms that explain its superior efficacy in reducing systemic inflammation and pain scores [38]. These strain-specific actions bridge our clinical findings, such as moderated pain scores and inflammation levels, to underlying biological processes, underscoring the importance of targeted probiotic selection in DD management [39].

### 4.2. Abdominal Bloating Improvement

Our review indicates that probiotics may help reduce abdominal bloating in patients with diverticular disease, although the overall effect size (0.158) was not statistically significant. This potential benefit could be explained by the capacity of probiotics to restore microbial balance, strengthen mucosal barrier function, and enhance bowel motility. Improvements in abdominal bloating were a consistent finding across most of the reviewed studies. For instance, *Bacillus coagulans* was identified as one of the most effective strains in reducing bloating in patients with irritable bowel syndrome [40]. This is supported by a meta-analysis of 26 randomized controlled trials (n = 2222), which reported a modest but statistically significant benefit of combination probiotics on bloating/distension [41]. Specifically, *B. coagulans* GBI-30, 6086 was found to be a safe and potentially effective probiotic for IBS-related bloating [42]. However, not all studies support the routine use of probiotics for bloating. Some trials have reported no significant differences in symptom relief, including bloating, when using probiotics such as VSL#3 in IBS patients [43]. Others have raised concerns about potential adverse effects, including bloating, brain fog, and even acidosis in susceptible individuals [44]. These discrepancies may be due to differences in study populations, probiotic strains, dosages, or outcome measures.

### 4.3. C-Reactive Protein

C-reactive protein (CRP) is a widely recognized biomarker of systemic inflammation and has been frequently used to evaluate the immunomodulatory effects of probiotics in gastrointestinal conditions, including diverticular disease (DD) [45]. Beyond DD, CRP has also shown prognostic value in patients with other gastrointestinal disorders. In inflammatory bowel disease (IBD), particularly Crohn’s disease, CRP levels correlate with disease activity [46], while in colorectal cancer (CRC), elevated CRP is associated with advanced stage and poor outcomes [47]. In the context of acute uncomplicated diverticulitis (AUD), CRP is especially valuable due to its association with disease severity, treatment response, and risk of complications. A threshold value of 175 mg/L was seen to exhibit a sensitivity of 61% and specificity of 82% and a negative predictive value of 92% for differentiation between complicated and uncomplicated disease [48]. Compared to other markers, CRP was found to be more reliable in the acute phase than white blood cell count or erythrocyte sedimentation rate [49]. However, its utility diminishes in less severe phenotypes, such as (SUDD), characterized by low-grade or intermittent inflammation. In these cases, it is well acknowledged that CRP can still be within normal limits, thus hampering its utility in both the diagnosis and the follow-up [24]. Fecal calprotectin, by contrast, has been proposed as a more sensitive marker for mucosal inflammation, correlating well with histologic activity and treatment response [35]. Nonetheless, its use remains controversial due to potential confounding factors such as medications or infections, and current guidelines do not endorse its routine application in DD [11]. Nonetheless, CRP is a useful endpoint in the acute setting.

In our review, CRP reductions were observed across the limited AUD studies evaluating probiotics, suggesting a potential anti-inflammatory effect consistent with experimental evidence indicating that probiotics may modulate cytokine responses and support mucosal immune balance [50]. However, in SUDD—where background inflammation is often mild—effects on CRP were moderate or unreported [32]. This raises concerns about CRP sensitivity in chronic disease and underscores the need for alternative markers (e.g., fecal calprotectin, IL-6, TNF-α) in future studies to evaluate probiotic effects on immune activity [51,52].

### 4.4. Probiotics & Prevention of Diverticular Disease Recurrence

Our results suggest that probiotics help prevent symptom recurrence. For example, Tursi et al. reported recurrence rates of 7.3% in the probiotic group versus 46.0% in controls, indicating a strong synergistic effect between the probiotic and the anti-inflammatory drug [25]. However, not all studies found consistent benefits. Kvasnovsky et al. reported no significant reduction in abdominal pain despite improvements in secondary symptoms, suggesting that probiotics may not alleviate all core symptoms but can still enhance overall quality of life [23].

These findings are consistent with literature on ileal Crohn’s disease, which has shown limited and inconclusive benefits from probiotic therapy. Some randomized trials have even reported higher relapse rates in probiotic-treated groups, although these differences were not statistically significant, likely due to underpowered sample sizes [53]. A similar pattern has been observed in patients with ulcerative colitis, where those who received probiotics tended to have fewer symptom recurrences compared to those who did not. While the results suggest a potential benefit, they were not conclusive [54]. Additionally, both individual RCTs, such as the one using *Lactobacillus* GG postoperatively, and larger meta-analyses suggest that probiotics alone do not significantly prevent recurrence in Crohn’s disease, though combinations with *E. coli* or *Saccharomyces* may hold future promise [55,56]. Collectively, these findings reflect a consistent trend: probiotics may offer modest clinical benefits, particularly for maintenance therapy, but the current evidence remains insufficient to confirm their efficacy with certainty.

Finally, the current major guidelines do not support the use of probiotics in diverticular disease. The American Gastroenterological Association (AGA) recommends against prescribing probiotics for preventing recurrence in patients with a history of diverticulitis due to insufficient evidence [57]. Similarly, the American Society of Colon and Rectal Surgeons (ASCRS) notes that although probiotics have been evaluated in several studies, the available data are inconsistent and do not clearly establish a preventive benefit [58]. National Institute for Health and Core Excellence (NICE) likewise reports that evidence for probiotics in this condition is very limited and does not justify their use for symptom management or modifying disease progression [59].

### 4.5. Limitations

One key limitation of our study is the high heterogeneity among included trials in terms of probiotic strains, dosages, treatment durations, and measured outcomes, which made data synthesis and direct comparisons challenging. Many studies used different combinations of bacterial species, making it difficult to isolate the effect of specific strains. Additionally, variation in study designs, such as randomized controlled trials versus observational studies, contributed to this heterogeneity. The limited number of high-quality RCTs, and their frequently small sample sizes, may have reduced the statistical power and increased the risk of errors. Furthermore, inconsistent diagnostic criteria for diverticular disease across studies may have introduced selection bias by including heterogeneous patient populations, potentially affecting the generalizability of the findings. Short follow-up durations in many studies limited the assessment of the long-term sustainability of probiotic effects, including prevention of recurrence. In addition, assessment of publication bias suggested that smaller or negative studies may be missing, with trim-and-fill adjustments yielding smaller effect sizes for several outcomes. Multiple-testing corrections also showed that some secondary findings did not retain statistical significance. Finally, inadequate blinding or allocation concealment in several studies increased the risk of performance and detection biases.

### 4.6. Recommendations

Based on our findings, we recommend standardizing future probiotic trials in diverticular disease by using consistent probiotic strains, dosages, treatment durations, and outcome measures. Research should also investigate strain-specific effects, as different probiotic species may yield varying results. Long-term, randomized controlled trials with larger sample sizes are essential to evaluate the impact of probiotics on recurrence rates and sustained symptom control. Additionally, future studies should minimize biases by employing robust study designs with proper randomization, blinding, and follow-up reporting to strengthen the evidence base

## 5. Conclusions

In conclusion, this meta-analysis indicates that probiotics are an effective adjuvant in managing diverticular disease. Strain specificity, treatment duration, and disease phenotype appear to influence their effectiveness in alleviating abdominal pain and systemic inflammation. While results are promising for both acute and chronic patients, inconsistencies across studies suggest that personalized, evidence-based probiotic strategies are needed. Future well-designed, large-scale RCTs should aim to standardize dosing, identify effective strains, and clarify biomarkers of response to guide personalized use of probiotics in clinical gastroenterology.

## Figures and Tables

**Table 1:** Included Studies Characteristics and Probiotic Intervention Details.

Study	Design	Country	Setting	Total N	Int/Ctrl N	Mean Age (Years)	Male (%)	Disease Type	Probiotic Strain (s)	Daily CFU	Duration	Schedule	Control Type
Ojetti et al. 2022 [19]	Double-blind RCT	Italy	Hospital ED	119	61/58	65.1 ± 20.0	41.2	AUD	*Limosilactobacillus reuteri* ATCC PTA 4659	5 × 10^8^	10 days	Twice daily	Placebo
Aragona et al. 2022 [20]	Clinical Experience	Italy	Multicenter	388	388 (pre-post)	62.9	53.1	SUDD/Symptomatic DD	Divercol^®^ (*L. rhamnosus*, *L. reuteri*, *L. acidophilus*—tyndallized)	3 × 10^9^	2 weeks	Daily	Pre-post design
Petruzziello et al. 2019 [21]	Double-blind RCT	Italy	Hospital ED	88	44/44	61.9 ± 13.9	38.6	AUD	*L. reuteri* ATCC PTA 4659	10^9^	10 days	Twice daily	Placebo
Petruzziello et al. 2019 [22]	Open-label controlled trial	Italy	Hospital ED	84	42/42	61.5 ± 11.5	29.8	AUD	Lactibiane Iki (*B. lactis* LA 304, *L. salivarius* LA 302, *L. acidophilus* LA 201)	8 × 10^10^	10 days	Twice daily	Antibiotics alone
Kvasnovsky et al. 2017 [23]	Double-blind RCT	UK	Tertiary hospital clinic	143	71/72	61.8 (median)	44.4	SUDD	Symprove (*L. rhamnosus* NCIMB 30174, *L. plantarum* NCIMB 30173, *L. acidophilus* NCIMB 30175, *E. faecium* NCIMB 30176)	10^10^	3 months	Daily	Placebo
Campanini et al. 2016 [24]	Prospective interventional	Italy	Primary Care	178	43/63	71.7 ± 11.5	32.2	SUDD	*Lactobacillus*/*Bifidobacterium* consortium	2.4 × 10^10^	3 months	20 days/month	Rifaximine alone
Tursi et al. 2013 [25]	Double-blind RCT	Italy	Multicenter	210	109/50	62.5 (median)	43.3	SUDD	*L. casei* DG	2.4 × 10^10^	12 months	10 days/month	Placebo
Lahner et al. 2012 [26]	Multicenter RCT	Italy	Multicenter	52	30/22	66.3 ± 9.5	32.7	SUDD	*L. paracasei* B21060	5 × 10^9^	6 months	Daily	High-fiber diet
Annibale et al. 2011 [27]	Pilot RCT	Italy	Multicenter Academic	50	34/16	65.2 ± 8.1	36.0	SUDD	*L. paracasei* F19	1.2–4.8 × 10^10^	6 months	14 days/month	High-fiber diet
Lamiki et al. 2010 [28]	Prospective open-label	Japan/Italy	Multicenter	46	46/0	62.5	21.7	SUDD (constipation)	SCM-III (*L. acidophilus* 145, *L. helveticus* ATC 15009, *Bifidobacterium* spp. 420)	~10^11^	6 months	Three times daily	Single-arm
Tursi et al. 2007 [29]	Pilot controlled trial	Italy	Multicenter	30	15/15	60.1	63.3	Uncomplicated diverticulitis	VSL#3 (8 strains: 4 *Lactobacillus*, 3 *Bifidobacterium*, 1 *Streptococcus*)	4.5 × 10^11^	12 months	15 days/month	Probiotic alone
Tursi et al. 2006 [30]	Prospective RCT	Italy	Multicenter	90	58/30	67.5	40.0	SUDD	*L. casei* DG	1.6 × 10^10^	12 months	15 days/month	Mesalazine
Fric et al. 2003 [31]	Prospective open trial	Czech Republic	Outpatient	15	15/0	74.8	33.3	SUDD	*E. coli* Nissle 1917	2.5–5.0 × 10^10^	5.2 weeks (avg)	Daily	Historical control

Abbreviations: AUD, Acute Uncomplicated Diverticulitis; CFU, Colony Forming Units; Ctrl, Control; DD, Diverticular Disease; ED, Emergency Department; Int, Intervention; N, Number of participants; RCT, Randomized Controlled Trial; SUDD, Symptomatic Uncomplicated Diverticular Disease.

**Table 2:** Primary and Secondary Outcomes Analysis.

Study/Analysis	Design	Disease	Probiotic	N (Int/Ctrl)	Pain Baseline (Int/Ctrl)	Pain Post-Treatment	Pain Reduction	Pain SMD (95% CI)	Bloating SMD (95% CI)	Notes
Ojetti et al. 2022 [19]	RCT	AUD	Single strain	61/58	7.0 ± NR/7.0 ± NR	0.0 ± NR/0.0 ± NR	7.0/7.0	NR (no SDs)	NR	Complete resolution both groups
Petruzziello et al. 2019 [21]	RCT	AUD	Single strain	44/44	8.2 ± 0.2/7.9 ± 0.3	0.13 ± 0.06/1.1 ± 0.25	8.07/6.80	4.019 (3.293, 4.745)	NR	Very large effect size
Petruzziello et al. 2019 [22] (second study)	RCT	AUD	Multi-strain	42/42	7.9 ± NR/8.0 ± NR	0.1 ± NR/0.8 ± NR	7.8/7.2	NR (no SDs)	NR	Substantial reduction both groups
AUD RCT SUBGROUP	Meta-analysis	1 study with complete data	-	-	-	-	-	4.019 (3.293, 4.745)	N/A	Very large effect for AUD
Kvasnovsky et al. 2017 [23]	RCT	SUDD	Multi-strain	71/72	3.4 ± 2.8/2.7 ± 2.5	2.1 ± 2.4/2.2 ± 2.3	1.3/0.5	0.225 (−0.103, 0.554)	0.137 (−0.191, 0.464)	Non-significant improvements
Tursi et al. 2013 [25]	RCT	SUDD	Single strain	109/50	0 (asymptomatic at baseline)	NR	NR	NR	NR	Prevention study, recurrence focus
Lahner et al. 2012 [26]	RCT	SUDD	Single strain	24/21	4.6 ± 2.2/4.6 ± 2.8	2.2 ± 0.8/2.0 ± 1.9	2.4/2.6	−0.068 (−0.654, 0.517)	−0.152 (−0.741, 0.437)	Control group improved more
Annibale et al. 2011 [27]	RCT	SUDD	Single strain	18/16	3.7 ± 3.5/2.8 ± 2.4	1.9 ± 2.2/2.3 ± 2.3	1.8/0.5	0.336 (−0.342, 1.014)	0.681 (−0.019, 1.382)	Moderate effects, wide CIs
Tursi et al. 2006 [30]	RCT	SUDD	Single strain	58/30	0 (asymptomatic at baseline)	NR	NR	NR	NR	Prevention study, recurrence focus
Fric et al. 2003 [31]	RCT	SUDD	Single strain	15 (historical control)	9.1 (historical)	1.2	7.9	NR (historical control)	NR (historical control)	Single-arm design
SUDD RCT SUBGROUP	Meta-analysis	3 studies with complete data	-	-	-	-	-	0.182 (−0.082, 0.446)	0.158 (−0.107, 0.422)	Small non-significant effects
		I^2^ = 10.3% (pain), 39.0% (bloating)	-	-	-	-	-	-	-	Heterogeneity assessment
ALL RCTs OVERALL	Meta-analysis	4 studies with complete pain data, 3 with bloating data	-	-	-	-	-	0.630 (0.382, 0.879)	0.158 (−0.107, 0.422)	Medium effect for pain, high heterogeneity
		I^2^ = 96.9% (pain), 39.0% (bloating)	-	-	-	-	-	-	-	Heterogeneity assessment
Non-Randomized Studies:										
Aragona et al. 2022 [20]	Non-RCT	SUDD	Multi-strain (tyndallized)	388 (single-arm)	3.7 ± NR	1.5 ± NR	2.2	NR (no control)	NR (no control)	Pre-post design, large sample
Campanini et al. 2016 [24]	Non-RCT	SUDD	Multi-strain	43/63	1.5 ± 1.0/NR	0.9 ± 0.8/NR	0.6/NR	NR (active comparator)	NR (active comparator)	Compared to rifaximine alone
Lamiki et al. 2010 [28]	Non-RCT	SUDD	Multi-strain	46 (single-arm)	NR	NR	NR	NR	NR	Outcomes not clearly reported
Tursi et al. 2007 [29]	Non-RCT	Uncomplicated diverticulitis	Multi-strain	15/15	NR	NR	NR	NR	NR	Pilot controlled trial, pain scores NR

Abbreviations: AUD, Acute Uncomplicated Diverticulitis; CI, Confidence Interval; Ctrl, Control; Int, Intervention; N, Number of participants; NR, Not Reported; RCT, Randomized Controlled Trial; SD, Standard Deviation; SMD, Standardized Mean Difference; SUDD, Symptomatic Uncomplicated Diverticular Disease.

**Table 3:** Inflammatory Biomarkers and Long-term Outcomes.

Study/Analysis	Design	Disease	Follow-Up	N (Int/Ctrl)	CRP Baseline (Int/Ctrl) mg/L	CRP Post-Treatment mg/L	CRP Reduction (Int/Ctrl)	CRP WMD (95% CI)	Recurrence Events (Int/Ctrl)	Recurrence Rate %	Risk Ratio (95% CI)	Notes
Inflammatory Biomarkers (AUD Studies):												
Ojetti et al. 2022 [19]	RCT	AUD	10 days	61/58	570.0 ± 225.0/377.1 ± 112.0	235.1 ± 100.5/226.0 ± 84.0	334.9/151.1	+183.8 (95.2, 272.4)	NR	NR	NR	Greater CRP reduction in intervention
Petruzziello et al. 2019 [21]	RCT	AUD	10 days	44/44	68.0 ± 8.8/71.3 ± 8.9	22.6 ± 4.0/43.8 ± 5.2	45.4/27.5	+17.9 (14.1, 21.7)	NR	NR	NR	Significant anti-inflammatory effect
Petruzziello et al. 2019 (1) [22]	RCT	AUD	10 days	42/42	764.4/679.0	274.6/461.6	489.8/217.4	+272.4 (no CI)	NR	NR	NR	Large reduction, SDs not reported
AUD CRP META-ANALYSIS	Meta-analysis	3 studies	10 days	147/144	-	-	-	Not pooled (range: +17.9 to +272.4)	-	-	-	Significant anti-inflammatory effect
		High heterogeneity due to baseline differences										Descriptive pooling only
Long-Term Outcomes (≥6 months):												
Tursi et al. 2013 [25]	RCT	SUDD	12 months	109/50	NR	NR	NR	NR	8/23	7.3/46.0	0.159 (0.077, 0.328)	84% reduction in recurrence
Tursi et al. 2006 [30]	RCT	SUDD	12 months	58/30	NR	NR	NR	NR	4/4	6.9/13.3	0.517 (0.140, 1.910)	Non-significant reduction
Lahner et al. 2012 [26]	RCT	SUDD	6 months	30/22	4.6 ± 2.2/4.6 ± 2.8	2.2 ± 0.8/2.0 ± 1.9	2.4/2.6	−0.2 (−1.1, 0.7)	0/0	0.0/0.0	NE (no events)	No recurrences in either group
SUDD RECURRENCE META-ANALYSIS	Meta-analysis	2 studies with events	12 months	167/80	-	-	-	-	12/27	7.2/33.8	0.220 (0.095, 0.510)	78% reduction in recurrence risk
		I^2^ = 45.2% (moderate heterogeneity)										*p* = 0.0005 (statistically significant)
Fric et al. 2003 [31]	RCT	SUDD	8–40 months	15 (historical)	9.1 (historical)	1.2	7.9	NR (historical control)	4/NR	26.7/NR	NR	Single-arm with historical comparison
Non-Randomized Studies:												
Lamiki et al. 2010 [28]	Non-RCT	SUDD	6 months	46 (single-arm)	NR	NR	NR	NR	14/NA	31.1/NA	NR	Constipation-predominant SUDD
Tursi et al. 2007 [29]	Non-RCT	Uncomplicated diverticulitis	12 months	15/15	NR	NR	NR	NR	1/2	6.7/13.3	0.500 (0.050, 5.030)	Pilot controlled trial
Campanini et al. 2016 [24]	Non-RCT	SUDD	3 months	43/63	NR	NR	NR	NR	NR	NR	NR	Active comparator design
Aragona et al. 2022 [20]	Non-RCT	SUDD	21 days	388 (single-arm)	NR	NR	NR	NR	NR	NR	NR	Pre-post design, short follow-up

Abbreviations: AUD, Acute Uncomplicated Diverticulitis; CI, Confidence Interval; CRP, C-Reactive Protein; Ctrl, Control; Int, Intervention; N, Number of participants; NE, Not Estimable; NR, Not Reported; RCT, Randomized Controlled Trial; RR, Risk Ratio; SUDD, Symptomatic Uncomplicated Diverticular Disease; WMD, Weighted Mean Difference.

**Table 4:** Safety and Adverse Events Profile.

Study/Analysis	Design	Disease	Duration	N (Int/Ctrl)	Total AEs (Int/Ctrl)	AE Rate % (Int/Ctrl)	Serious AEs (Int/Ctrl)	Withdrawals due to AEs (Int/Ctrl)	Total Withdrawals (Int/Ctrl)	Risk Ratio (95% CI)	AE Details	Notes
Acute Studies (≤2 weeks):												
Ojetti et al. 2022 [19]	RCT	AUD	10 days	61/58	0/0	0.0/0.0	0/0	0/0	0/0	NE	None reported	Complete safety profile
Petruzziello et al. 2019 [21]	RCT	AUD	10 days	44/44	0/0	0.0/0.0	0/0	0/0	0/0	NE	None reported	>95% compliance
Petruzziello et al. 2019 (1) [22]	RCT	AUD	10 days	42/42	0/0	0.0/0.0	0/0	0/0	0/0	NE	None reported	>95% compliance
Aragona et al. 2022 [20]	Non-RCT	SUDD	2 weeks	388	0	0.0	0	0	0	NE	None reported	“Safely tolerated”
ACUTE STUDIES META-ANALYSIS	Meta-analysis	AUD/SUDD	≤2 weeks	535/144	0/0	0.0/0.0	0/0	0/0	0/0	NE	-	Excellent short-term safety
Intermediate Studies (1–6 months):												
Kvasnovsky et al. 2017 [23]	RCT	SUDD	3 months	71/72	15/13	21.1/18.1	0/0	8/2	15/8	1.17 (0.64, 2.13)	Int: nausea, reflux, constipation; Ctrl: cramps, bleeding, bloating	Most AEs mild, GI-related
Campanini et al. 2016 [24]	Non-RCT	SUDD	3 months	43/63	NR/NR	NR/NR	NR/NR	NR/NR	4/7	NR	Not systematically reported	Active comparator design
Lahner et al. 2012 [26]	RCT	SUDD	6 months	30/22	3/0	10.0/0.0	0/0	3/0	10/1	5.13 (0.28, 93.02)	Int: 1 constipation, 2 worse pain	Higher AE rate in probiotic group
Lamiki et al. 2010 [28]	Non-RCT	SUDD	6 months	46	4	8.7	0	0	1	NE	4 poor palatability (9%)	>98% compliance despite taste
Annibale et al. 2011 [27]	RCT	SUDD	6 months	34/16	1/0	2.9/0.0	0/0	1/0	4/1	1.41 (0.06, 33.27)	Int: 1 diarrhea	Mild GI symptoms only
INTERMEDIATE STUDIES META-ANALYSIS	Meta-analysis	SUDD	3–6 months	135/110	19/13	14.1/11.8	0/0	12/2	29/9	1.32 (0.73, 2.39)	-	Mild GI AEs, no serious events
Long-Term Studies (≥12 months):												
Tursi et al. 2013 [25]	RCT	SUDD	12 months	109/50	0/0	0.0/0.0	0/0	0/0	0/0	NE	None related to study drugs	Excellent long-term tolerance
Tursi et al. 2006 [30]	RCT	SUDD	12 months	58/30	2/1	3.4/3.3	0/0	0/0	1/1	1.03 (0.10, 10.98)	Int: epigastric pain, nausea; Ctrl: epigastric pain	Mild GI symptoms
Tursi et al. 2007 [29]	Non-RCT	Uncomplicated diverticulitis	12 months	15/15	0/0	0.0/0.0	0/0	0/0	1/1	NE	None reported	Withdrawals due to compliance issues
Fric et al. 2003 [31]	RCT	SUDD	5.2 weeks	15	0	0.0	0	0	0	NE	No undesired side effects	Historical control design
LONG-TERM STUDIES META-ANALYSIS	Meta-analysis	SUDD	≥12 months	167/80	2/1	1.2/1.3	0/0	0/0	1/1	0.87 (0.08, 9.32)	-	Excellent long-term safety
Overall Safety Profile:												
ALL RCTs POOLED	Meta-analysis	All diseases	Variable	464/334	21/14	4.5/4.2	0/0	12/2	30/11	1.07 (0.58, 1.99)	-	Similar safety to controls

Abbreviations: AE, Adverse Event; AUD, Acute Uncomplicated Diverticulitis; CI, Confidence Interval; Ctrl, Control; GI, Gastrointestinal; Int, Intervention; N, Number of participants; NE, Not Estimable; NR, Not Reported; RCT, Randomized Controlled Trial; RR, Risk Ratio; SUDD, Symptomatic Uncomplicated Diverticular Disease.

**Table 5:** Meta-Regression and Sensitivity Analysis.

Analysis Type	Specific Analysis	Studies/Criteria	Sample Size	Statistical Measure	Effect Size/Coefficient	95% CI	R^2^/I^2^ (%)	*p*-Value	Change From Baseline
Meta-Regression:									
Dose–Response	Pain reduction	Log_10_ CFU count	4 studies	β coefficient	−2.651	−5.638, 0.336	R^2^ = 64.6	≥0.05	N/A
Treatment Duration	Pain reduction	Duration (days)	4 studies	β coefficient	−0.016	−0.033, 0.002	R^2^ = 47.8	≥0.05	N/A
Study Quality	Pain reduction	Quality score (1–10)	4 studies	β coefficient	0.392	−0.323, 1.107	R^2^ = 5.2	≥0.05	N/A
Disease Severity	Pain reduction	AUD vs. SUDD	4 studies	β coefficient	3.855	2.022, 5.688	R^2^ = 89.1	<0.001	N/A
Recurrence Prevention	Recurrence rate	Log_10_ CFU count	2 studies	β coefficient	NE	NE	NE	NE	N/A
Sensitivity Analysis:									
Baseline Analysis	All eligible RCTs	None excluded	4 studies	SMD	0.630	0.382, 0.879	I^2^ = 96.9	<0.05	Reference
Study Quality	Exclude low quality	Quality score < 7	3 studies	SMD	0.669	0.394, 0.944	I^2^ = 97.2	<0.05	+6.2%
Sample Size	Exclude small studies	Studies n < 50	4 studies	SMD	0.624	0.375, 0.873	I^2^ = 96.8	<0.05	−0.9%
Treatment Duration	Exclude short duration	Duration < 30 days	3 studies	SMD	0.182	−0.082, 0.446	I^2^ = 10.3	≥0.05	−71.1%
Combined Exclusions	Quality < 7 + n < 50	Both criteria	3 studies	SMD	0.669	0.394, 0.944	I^2^ = 97.2	<0.05	+6.2%
Model Comparison:									
Fixed Effects	All studies	Standard model	4 studies	SMD	3.515	3.370, 3.660	I^2^ = 96.9	<0.001	+457.8%
Random Effects	All studies	Preferred model	4 studies	SMD	0.630	0.382, 0.879	I^2^ = 96.9	<0.05	Reference
Influence Analysis:									
Remove Petruzziello 2019 [21]	Largest effect study	AUD study excluded	3 studies	SMD	0.182	−0.082, 0.446	I^2^ = 10.3	≥0.05	−71.1%
Remove Kvasnovsky 2017 [23]	Largest sample study	Multi-strain excluded	3 studies	SMD	0.741	0.421, 1.061	I^2^ = 96.8	<0.001	+17.6%
Remove Lahner 2012 [26]	Negative effect study	Smallest effect excluded	3 studies	SMD	0.856	0.512, 1.200	I^2^ = 97.1	<0.001	+35.9%
Remove Annibale 2011 [27]	Smallest sample study	Pilot study excluded	3 studies	SMD	0.615	0.358, 0.872	I^2^ = 97.0	<0.05	−2.4%
Heterogeneity Exploration:									
AUD Subgroup	Disease-specific	Acute studies only	1 study	SMD	4.019	3.293, 4.745	I^2^ = N/A	<0.001	+537.9%
SUDD Subgroup	Disease-specific	Chronic studies only	3 studies	SMD	0.182	−0.082, 0.446	I^2^ = 10.3	≥0.05	−71.1%
Between-Group Test	AUD vs. SUDD	Disease comparison	4 studies	Difference	3.837	2.945, 4.729	Q = 89.1	<0.001	N/A
Single Strain	Probiotic type	Mono-bacterial	3 studies	SMD	0.420	0.141, 0.699	I^2^ = 52.1	<0.05	−33.3%
Multi-strain	Probiotic type	Poly-bacterial	1 study	SMD	0.225	−0.103, 0.554	I^2^ = N/A	≥0.05	−64.3%
Strain Comparison	Single vs. Multi	Probiotic comparison	4 studies	Difference	0.195	−0.432, 0.822	Q = 8.7	0.56	N/A

Abbreviations: AUD, Acute Uncomplicated Diverticulitis; CI, Confidence Interval; NE, Not Estimable; Q, Cochran’s Q statistic; SMD, Standardized Mean Difference; SUDD, Symptomatic Uncomplicated Diverticular Disease.

## Data Availability

All relevant data supporting the findings of this study are included within the manuscript.

## Supplementary Materials

The following supporting information can be downloaded at: https://www.mdpi.com/article/10.3390/jcm15010088/s1, Table S1: Search strategy used in each of the databases; Table S2: PRISMA-2020 checklist; Table S3: Risk of bias assessment; Table S4: Certainty assessment of evidence.

## Abbreviations

The following abbreviations are used in this manuscript:

PRISMA	Preferred Reporting Items for Systematic reviews and Meta-Analyses
MeSH	Medical Subject Headings
DD	Diverticular Disease
RCTs	Randomized Controlled Trials
SMD	Standardized Mean Difference
CI	Confidence Interval
CRP	C-reactive Protein
RR	Relative Risk
SCAD	Segmental Colitis associated with Diverticulosis
SUDD	Symptomatic Uncomplicated Diverticular Disease
CT	Computed Tomography
CTC	Computed Tomography Colonography
CDD	Classification of Diverticular Disease
CENTRAL	Cochrane Central Register of Controlled Trials
AUD	Acute Uncomplicated Diverticulitis
RoB 2.0	Risk of Bias
ROBINS-I	Risk of Bias in Non-Randomized Studies of Interventions
GRADE	The Grading of Recommendations Assessment, Development, and Evaluation
FDR	False Discovery Rate
IBS	Irritable Bowel Syndrome
IBD	Inflammatory Bowel Disease
AGA	American Gastroenterological Association
ASCRS	American Society of Colon and Rectal Surgeons
NICE	National Institute for Health and Core Excellence

## References

[1] Tursi A. (2016). Diverticulosis Today: Unfashionable and Still under-Researched. Ther. Adv. Gastroenterol..

[2] Yang F., Sun X., Jiang K. (2025). Distribution and Characteristics of Colonic Diverticula in Northern China. J. Clin. Gastroenterol..

[3] Azzam N. (2013). Prevalence and Clinical Features of Colonic Diverticulosis in a Middle Eastern Population. World J. Gastrointest. Endosc..

[4] Carabotti M., Sgamato C., Amato A., Beltrame B., Binda G.A., Germanà B., Leandro G., Pasquale L., Peralta S., Viggiani M.T. (2024). Italian Guidelines for the Diagnosis and Management of Colonic Diverticulosis and Diverticular Disease. Dig. Liver Dis..

[5] Cuomo R., Barbara G., Pace F., Annese V., Bassotti G., Binda G.A., Casetti T., Colecchia A., Festi D., Fiocca R. (2014). Italian Consensus Conference for Colonic Diverticulosis and Diverticular Disease. United Eur. Gastroenterol. J..

[6] Calini G., Abd El Aziz M.A., Paolini L., Abdalla S., Rottoli M., Mari G., Larson D.W. (2023). Symptomatic Uncomplicated Diverticular Disease (SUDD): Practical Guidance and Challenges for Clinical Management. Clin. Exp. Gastroenterol..

[7] Buldukoğlu O.Ç., Öcal S., Atar G.E., Harmandar F.A., Çekin A.H. (2024). Sit or Squat? Toilet Type Is a Determinant of Diverticulosis Development. Turk. J. Gastroenterol..

[8] Imaeda H., Hibi T. (2018). The Burden of Diverticular Disease and Its Complications: West versus East. Inflamm. Intest. Dis..

[9] Trifan A., Gheorghe C., Sabo C.M., Diculescu M., Nedelcu L., Singeap A.M., Sfarti C., Gheorghe L., Sporea I., Tanțău M. (2018). Diagnosis and Treatment of Colonic Diverticular Disease: Position Paper of the Romanian Society of Gastroenterology and Hepatology. J. Gastrointest. Liver Dis..

[10] Bhatia M., Mattoo A. (2023). Diverticulosis and Diverticulitis: Epidemiology, Pathophysiology, and Current Treatment Trends. Cureus.

[11] Kruis W., Germer C., Böhm S., Dumoulin F.L., Frieling T., Hampe J., Keller J., Kreis M.E., Meining A., Labenz J. (2022). German Guideline Diverticular Disease/Diverticulitis: Part II: Conservative, Interventional and Surgical Management. United Eur. Gastroenterol. J..

[12] Lock J.F., Galata C., Reißfelder C., Ritz J.-P., Schiedeck T., Germer C.-T. (2020). The Indications for and Timing of Surgery for Diverticular Disease. Dtsch. Ärzteblatt Int..

[13] Gross M., Beckenbauer U.E., Bruder L., Zehrer A. (2022). Divertikelkrankheit: Patientenmanagement in hausärztlichen Praxen in Deutschland: Hoher Stellenwert von Probiotika in der hausärztlichen Versorgung. MMW—Fortschritte Der Med..

[14] Page M.J., McKenzie J.E., Bossuyt P.M., Boutron I., Hoffmann T.C., Mulrow C.D., Shamseer L., Tetzlaff J.M., Akl E.A., Brennan S.E. (2021). The PRISMA 2020 Statement: An Updated Guideline for Reporting Systematic Reviews. BMJ.

[15] Alnajjar J., Alabdullatif N., Almutairi A., ALBohassan M., Almarzooq M., Alshafei A. (2024). Protocol for the Impact of Probiotics on Clinical Outcomes in Diverticular Disease: A Systematic Review and Meta-Analysis.

[16] Sterne J.A.C., Savović J., Page M.J., Elbers R.G., Blencowe N.S., Boutron I., Cates C.J., Cheng H.-Y., Corbett M.S., Eldridge S.M. (2019). RoB 2: A Revised Tool for Assessing Risk of Bias in Randomised Trials. BMJ.

[17] Sterne J.A., Hernán M.A., Reeves B.C., Savović J., Berkman N.D., Viswanathan M., Henry D., Altman D.G., Ansari M.T., Boutron I. (2016). ROBINS-I: A Tool for Assessing Risk of Bias in Non-Randomised Studies of Interventions. BMJ.

[18] GRADE|Cochrane. https://www.cochrane.org/learn/courses-and-resources/cochrane-methodology/grade.

[19] Ojetti V., Saviano A., Brigida M., Petruzziello C., Caronna M., Gayani G., Franceschi F. (2022). Randomized Control Trial on the Efficacy of *Limosilactobacillus reuteri* ATCC PTA 4659 in Reducing Inflammatory Markers in Acute Uncomplicated Diverticulitis. Eur. J. Gastroenterol. Hepatol..

[20] Aragona S.E., Pasquale L., Fedeli P., Galloro G., Carrara P., Massa D., Iannuzziello D., Paiano P., Savarino E., Vinti M. (2022). Diverticular Disease: A New Therapeutic Opportunity. J. Biol. Regul. Homeost. Agents.

[21] Petruzziello C., Migneco A., Cardone S., Covino M., Saviano A., Franceschi F., Ojetti V. (2019). Supplementation with *Lactobacillus reuteri* ATCC PTA 4659 in Patients Affected by Acute Uncomplicated Diverticulitis: A Randomized Double-Blind Placebo Controlled Trial. Int. J. Color. Dis..

[22] Petruzziello C., Marannino M., Migneco A., Brigida M., Saviano A., Piccioni A., Franceschi F., Ojetti V. (2019). The Efficacy of a Mix of Three Probiotic Strains in Reducing Abdominal Pain and Inflammatory Biomarkers in Acute Uncomplicated Diverticulitis. Eur. Rev. Med. Pharmacol. Sci..

[23] Kvasnovsky C.L., Bjarnason I., Donaldson A.N., Sherwood R.A., Papagrigoriadis S. (2017). A Randomized Double-Blind Placebo-Controlled Trial of a Multi-Strain Probiotic in Treatment of Symptomatic Uncomplicated Diverticular Disease. Inflammopharmacol.

[24] Campanini A., De Conto U., Cavasin F., Bastiani F., Camarotto A., Gardini L., Geremia A., Marastoni C., Missorini C., Quarantelli E. (2016). A Primary-Care Interventional Model on the Diverticular Disease: Searching for the Optimal Therapeutic Schedule. J. Clin. Gastroenterol..

[25] Tursi A., Brandimarte G., Elisei W., Picchio M., Forti G., Pianese G., Rodino S., D’Amico T., Sacca N., Portincasa P. (2013). Randomised Clinical Trial: Mesalazine and/or Probiotics in Maintaining Remission of Symptomatic Uncomplicated Diverticular Disease—A Double-Blind, Randomised, Placebo-Controlled Study. Aliment. Pharmacol. Ther..

[26] Lahner E. (2012). High-Fibre Diet and *Lactobacillus paracasei* B21060 in Symptomatic Uncomplicated Diverticular Disease. World J. Gastroenterol. WJG.

[27] Annibale B., Maconi G., Lahner E., De Giorgi F., Cuomo R. (2011). Efficacy of *Lactobacillus paracasei* Sub. Paracasei F19 on Abdominal Symptoms in Patients with Symptomatic Uncomplicated Diverticular Disease: A Pilot Study. Minerva Gastroenterol. Dietol..

[28] Lamiki P., Tsuchiya J., Pathak S., Okura R., Solimene U., Jain S., Kawakita S., Marotta F. (2010). Probiotics in Diverticular Disease of the Colon: An Open Label Study. J. Gastrointest. Liver Dis..

[29] Tursi A., Brandimarte G., Giorgetti G.M., Elisei W., Aiello F. (2007). Balsalazide and/or High-Potency Probiotic Mixture (VSL#3) in Maintaining Remission after Attack of Acute, Uncomplicated Diverticulitis of the Colon. Int. J. Color. Dis..

[30] Tursi A., Brandimarte G., Giorgetti G.M., Elisei W. (2006). Mesalazine and/or *Lactobacillus casei* in Preventing Recurrence of Symptomatic Uncomplicated Diverticular Disease of the Colon: A Prospective, Randomized, Open-Label Study. J. Clin. Gastroenterol..

[31] Fric P., Zavoral M. (2003). The Effect of Non-Pathogenic Escherichia Coli in Symptomatic Uncomplicated Diverticular Disease of the Colon. Eur. J. Gastroenterol. Hepatol..

[32] Marasco G., Buttitta F., Cremon C., Barbaro M.R., Stanghellini V., Barbara G. (2023). The Role of Microbiota and Its Modulation in Colonic Diverticular Disease. Neurogastroenterol. Motil..

[33] Bretto E., D’Amico F., Fiore W., Tursi A., Danese S. (2022). *Lactobacillus paracasei CNCM I* 1572: A Promising Candidate for Management of Colonic Diverticular Disease. J. Clin. Med..

[34] Tursi A., Papa V., Lopetuso L.R., Settanni C.R., Gasbarrini A., Papa A. (2022). Microbiota Composition in Diverticular Disease: Implications for Therapy. Int. J. Mol. Sci..

[35] Lahner E., Bellisario C., Hassan C., Zullo A., Esposito G., Annibale B. (2016). Probiotics in the Treatment of Diverticular Disease. A systematic review. J. Gastrointest. Liver Dis..

[36] Aghamohammad S., Sepehr A., Miri S.T., Najafi S., Rohani M., Pourshafiea M.R. (2022). The effects of the probiotic cocktail on modulation of the NF-kB and JAK/STAT signaling pathways involved in the inflammatory response in bowel disease model. BMC Immunol..

[37] Barbara G., Stanghellini V., Brandi G., Cremon C., Di Nardo G., De Giorgio R., Corinaldesi R. (2005). Interactions between commensal bacteria and gut sensorimotor function in health and disease. Am. J. Gastroenterol..

[38] Gao C., Major A., Rendon D., Lugo M., Jackson V., Shi Z., Mori-Akiyama Y., Versalovic J. (2015). Histamine H2 Receptor-Mediated Suppression of Intestinal Inflammation by Probiotic *Lactobacillus reuteri*. mBio.

[39] Piccioni A., Franza L., Vaccaro V., Saviano A., Zanza C., Candelli M., Covino M., Franceschi F., Ojetti V. (2021). Microbiota and Probiotics: The Role of *Limosilactobacillus reuteri* in Diverticulitis. Medicina.

[40] Zhang T., Zhang C., Zhang J., Sun F., Duan L. (2022). Efficacy of Probiotics for Irritable Bowel Syndrome: A Systematic Review and Network Meta-Analysis. Front. Cell. Infect. Microbiol..

[41] Goodoory V.C., Khasawneh M., Black C.J., Quigley E.M.M., Moayyedi P., Ford A.C. (2023). Efficacy of Probiotics in Irritable Bowel Syndrome: Systematic Review and Meta-analysis. Gastroenterology.

[42] Hun L. (2009). Original Research: *Bacillus coagulans* Significantly Improved Abdominal Pain and Bloating in Patients with IBS. Postgrad. Med..

[43] Kim H.J., Vazquez Roque M.I., Camilleri M., Stephens D., Burton D.D., Baxter K., Thomforde G., Zinsmeister A.R. (2005). A Randomized Controlled Trial of a Probiotic Combination VSL# 3 and Placebo in Irritable Bowel Syndrome with Bloating. Neurogastroenterol. Motil..

[44] Moshiree B., Drossman D., Shaukat A. (2023). AGA Clinical Practice Update on Evaluation and Management of Belching, Abdominal Bloating, and Distention: Expert Review. Gastroenterology.

[45] Mazidi M., Rezaie P., Ferns G., Vatanparast H. (2017). Impact of Probiotic Administration on Serum C-Reactive Protein Concentrations: Systematic Review and Meta-Analysis of Randomized Control Trials. Nutrients.

[46] Vermeire S. (2006). Laboratory Markers in IBD: Useful, Magic, or Unnecessary Toys?. Gut.

[47] McMillan D.C. (2013). The Systemic Inflammation-Based Glasgow Prognostic Score: A Decade of Experience in Patients with Cancer. Cancer Treat. Rev..

[48] Van De Wall B.J.M., Draaisma W.A., Van Der Kaaij R.T., Consten E.C.J., Wiezer M.J., Broeders I.A.M.J. (2013). The Value of Inflammation Markers and Body Temperature in Acute Diverticulitis. Color. Dis..

[49] Kumarasinghe D., Zahid A., O’Grady G., Leow T.Y., Sheriff T., Ctercteko G., Gosselink M., Adusumilli S. (2021). The Use of Biochemical Markers in Complicated and Uncomplicated Acute Diverticulitis. Int. Surg..

[50] Morreale C., Bresesti I., Bosi A., Baj A., Giaroni C., Agosti M., Salvatore S. (2022). Microbiota and Pain: Save Your Gut Feeling. Cells.

[51] Barbara G., Scaioli E., Barbaro M.R., Biagi E., Laghi L., Cremon C., Marasco G., Colecchia A., Picone G., Salfi N. (2017). Gut Microbiota, Metabolome and Immune Signatures in Patients with Uncomplicated Diverticular Disease. Gut.

[52] Hegazy S.K. (2010). Effect of Probiotics on Pro-Inflammatory Cytokines and NF-κB Activation in Ulcerative Colitis. World J. Gastroenterol. WJG.

[53] Cabré E., Gassull M.A. (2007). Probiotics for Preventing Relapse or Recurrence in Crohn’s Disease Involving the Ileum: Are There Reasons for Failure?. J. Crohn’s Colitis.

[54] Sang L.-X., Chang B., Zhang W.-L., Wu X.-M., Li X.-H., Jiang M. (2010). Remission Induction and Maintenance Effect of Probiotics on Ulcerative Colitis: A Meta-Analysis. World J. Gastroenterol..

[55] Rahimi R., Nikfar S., Rahimi F., Elahi B., Derakhshani S., Vafaie M., Abdollahi M. (2008). A Meta-Analysis on the Efficacy of Probiotics for Maintenance of Remission and Prevention of Clinical and Endoscopic Relapse in Crohn’s Disease. Dig. Dis. Sci..

[56] Prantera C., Scribano M.L., Falasco G., Andreoli A., Luzi C. (2002). Ineffectiveness of Probiotics in Preventing Recurrence after Curative Resection for Crohn’s Disease: A Randomised Controlled Trial with *Lactobacillus* GG. Gut.

[57] Peery A.F., Shaukat A., Strate L.L. (2021). AGA Clinical Practice Update on Medical Management of Colonic Diverticulitis: Expert Review. Gastroenterology.

[58] Feingold J.L., Steele S.R., Lee S., Kaiser A., Boushey R., Buie W.D., Rafferty J.F. (2020). Practice Parameters for the Treatment of Sigmoid Diverticulitis. Dis. Colon. Rectum.

[59] National Institute for Health and Care Excellence (NICE) (2019). Diverticular Disease: Diagnosis and Management (NG147). https://www.nice.org.uk/guidance/ng147.

