# Supplementary material for: The Impact of Probiotics on Clinical Outcomes in Diverticular Disease: A Systematic Review and Meta-Analysis
Source: J Clin Med. 2025 Dec 23;15(1):88. doi: 10.3390/jcm15010088 (PMC12786911; doi:10.3390/jcm15010088)
Supplement: Supplementary file 1 [file jcm-15-00088-s001.zip › jcm-3962262-supplementary.pdf]

Table S1. Search strategy used in each of the databases.

Data base	Search strategy	Number of Articles
Pubmed	("Rectal Prolapse"[Mesh] OR "Anus"[Mesh] AND "Prolapse"[Mesh] OR rectal prolapse[tiab] OR prolapse, rectal[tiab] OR rectal prolapses[tiab] OR anus prolapse[tiab] OR prolapse, anus[tiab])<br>AND<br>("Altemeier Procedure"[Supplementary Concept] OR Altemeier procedure[tiab] OR Altemeier's procedure[tiab] OR perineal rectosigmoidectomy[tiab] OR Altemeier perineal proctosigmoidectomy[tiab])	5267
Scopus	<1974 to 2024 Week 51><br><br>(TITLE-ABS-KEY("Altemeier's Procedure" OR "Altemeier perineal proctosigmoidectomy" OR "Altemeier procedure" OR "Perineal rectosigmoidectomy"))<br>AND<br>(TITLE-ABS-KEY("Rectal Prolapse" OR "Prolapse, Rectal" OR "Prolapses, Rectal" OR "Rectal Prolapses" OR "Anus Prolapse" OR "Anus Prolapses" OR "Prolapse, Anus" OR "Prolapses, Anus"))	195
Embase	("Altemeier's Procedure".mp. OR "Altemeier perineal proctosigmoidectomy".mp. OR "Altemeier procedure".mp. OR "Perineal rectosigmoidectomy".mp.)<br>AND<br>("Rectal Prolapse".mp. OR "Prolapse, Rectal".mp. OR "Prolapses, Rectal".mp. OR "Rectal Prolapses".mp. OR "Anus Prolapse".mp. OR "Anus Prolapses".mp. OR "Prolapse, Anus".mp. OR "Prolapses, Anus".mp.)	219
Cochrane	([mh "Altemeier Procedure"] OR "Altemeier's Procedure" OR "Altemeier perineal proctosigmoidectomy" OR "Altemeier procedure" OR "Perineal rectosigmoidectomy")<br>AND	10

	([mh "Rectal Prolapse"] OR "Rectal Prolapse" OR "Prolapse, Rectal" OR "Prolapses, Rectal" OR "Rectal Prolapses" OR "Anus Prolapse" OR "Anus Prolapses" OR "Prolapse, Anus" OR "Prolapses, Anus")	
Web of science	TS=("Altemeier's Procedure" OR "Altemeier perineal proctosigmoidectomy" OR "Altemeier procedure" OR "Perineal rectosigmoidectomy")<br>AND<br>TS=("Rectal Prolapse" OR "Prolapse, Rectal" OR "Prolapses, Rectal" OR "Rectal Prolapses" OR "Anus Prolapse" OR "Anus Prolapses" OR "Prolapse, Anus" OR "Prolapses, Anus")	175

Table S2. PRISMA 2020 Checklist

Section and Topic	Item #	Checklist item	Location where item is reported
**TITLE**			
Title	1	Identify the report as a systematic review.	Title page 1
**ABSTRACT**			
Abstract	2	See the PRISMA 2020 for Abstracts checklist.	Page 1
**INTRODUCTION**			
Rationale	3	Describe the rationale for the review in the context of existing knowledge.	Page 2
Objectives	4	Provide an explicit statement of the objective(s) or question(s) the review addresses.	Page 2
**METHODS**			
Eligibility criteria	5	Specify the inclusion and exclusion criteria for the review and how studies were grouped for the syntheses.	Pages 3-4
Information sources	6	Specify all databases, registers, websites, organizations, reference lists and other sources searched or consulted to identify studies. Specify the date when each source was last searched or consulted.	Page 3
Search strategy	7	Present the full search strategies for all databases, registers and websites, including any filters and limits used.	Pages 3
Selection process	8	Specify the methods used to decide whether a study met the inclusion criteria of the review, including how many reviewers screened each record and each report retrieved, whether they worked independently, and if applicable, details of automation tools used in the process.	Pages 4-5
Data collection process	9	Specify the methods used to collect data from reports, including how many reviewers collected data from each report, whether they worked independently, any processes for obtaining or confirming data from study investigators, and if applicable, details of automation tools used in the process.	Page 5
Data items	10a	List and define all outcomes for which data were sought. Specify whether all results that were compatible with each outcome domain in each study were sought (e.g. for all measures, time points, analyses), and if not, the methods used to decide which results to collect.	Pages 5
	10b	List and define all other variables for which data were sought (e.g. participant and intervention characteristics, funding sources). Describe any assumptions made about any missing or unclear information.	Page 5
Study risk of bias assessment	11	Specify the methods used to assess risk of bias in the included studies, including details of the tool(s) used, how many reviewers assessed each study and whether they worked independently, and if applicable, details of automation tools used in the process.	Page 5-6
Effect measures	12	Specify for each outcome the effect measure(s) (e.g. risk ratio, mean difference) used in the synthesis or presentation of results.	Page 5-6
Synthesis methods	13a	Describe the processes used to decide which studies were eligible for each synthesis (e.g. tabulating the study intervention characteristics and comparing against the planned groups for each synthesis (item #5)).	Page 6
	13b	Describe any methods required to prepare the data for presentation or synthesis, such as handling of missing summary statistics, or data conversions.	Page 6
	13c	Describe any methods used to tabulate or visually display results of individual studies and syntheses.	Page 6

Table S2. PRISMA 2020 Checklist

Section and Topic	Item #	Checklist item	Location where item is reported
	13d	Describe any methods used to synthesize results and provide a rationale for the choice(s). If meta-analysis was performed, describe the model(s), method(s) to identify the presence and extent of statistical heterogeneity, and software package(s) used.	Page 7-8
	13e	Describe any methods used to explore possible causes of heterogeneity among study results (e.g. subgroup analysis, meta-regression).	Page 7-8
	13f	Describe any sensitivity analyses conducted to assess robustness of the synthesized results.	Page 7-8
Reporting bias assessment	14	Describe any methods used to assess risk of bias due to missing results in a synthesis (arising from reporting biases).	Page 7
Certainty assessment	15	Describe any methods used to assess certainty (or confidence) in the body of evidence for an outcome.	Page 7-8
**RESULTS**			
Study selection	16a	Describe the results of the search and selection process, from the number of records identified in the search to the number of studies included in the review, ideally using a flow diagram.	Page 8-9, Figure 1 (Page 5)
	16b	Cite studies that might appear to meet the inclusion criteria, but which were excluded, and explain why they were excluded.	Page 8-9, Figure 1 (Page 5)
Study characteristics	17	Cite each included study and present its characteristics.	Pages 8-9, Tables 1 (Pages 9)
Risk of bias in studies	18	Present assessments of risk of bias for each included study.	Supplementary materials
Results of individual studies	19	For all outcomes, present, for each study: (a) summary statistics for each group (where appropriate) and (b) an effect estimate and its precision (e.g. confidence/credible interval), ideally using structured tables or plots.	Pages 10-21, Tables 2-5 (Pages 11-17).
Results of syntheses	20a	For each synthesis, briefly summarise the characteristics and risk of bias among contributing studies.	Page 19-20, Table 6
	20b	Present results of all statistical syntheses conducted. If meta-analysis was done, present for each the summary estimate and its precision (e.g. confidence/credible interval) and measures of statistical heterogeneity. If comparing groups, describe the direction of the effect.	Pages 12-21, Figures (2-6)
	20c	Present results of all investigations of possible causes of heterogeneity among study results.	Pages 18-21, Figure 5, Table 6
	20d	Present results of all sensitivity analyses conducted to assess the robustness of the synthesized results.	Pages 18-21
Reporting biases	21	Present assessments of risk of bias due to missing results (arising from reporting biases) for each synthesis assessed.	Page 19-20
Certainty of	22	Present assessments of certainty (or confidence) in the body of evidence for each outcome assessed.	Supplementary

Table S2. PRISMA 2020 Checklist

Section and Topic	Item #	Checklist item	Location where item is reported
evidence			materials
**DISCUSSION**			
Discussion	23a	Provide a general interpretation of the results in the context of other evidence.	Pages 21-23
	23b	Discuss any limitations of the evidence included in the review.	Pages 23
	23c	Discuss any limitations of the review processes used.	Pages 23
	23d	Discuss implications of the results for practice, policy, and future research.	Pages 21-23
**OTHER INFORMATION**			
Registration and protocol	24a	Provide registration information for the review, including register name and registration number, or state that the review was not registered.	Page 2
	24b	Indicate where the review protocol can be accessed, or state that a protocol was not prepared.	Page 2
	24c	Describe and explain any amendments to information provided at registration or in the protocol.	No amendments
Support	25	Describe sources of financial or non-financial support for the review, and the role of the funders or sponsors in the review.	Page 24
Competing interests	26	Declare any competing interests of review authors.	Page 24
Availability of data, code and other materials	27	Report which of the following are publicly available and where they can be found: template data collection forms; data extracted from included studies; data used for all analyses; analytic code; any other materials used in the review.	Page 24

From: Page MJ, McKenzie JE, Bossuyt PM, Boutron I, Hoffmann TC, Mulrow CD, et al. The PRISMA 2020 statement: an updated guideline for reporting systematic reviews. BMJ 2021;372:n71. doi: 10.1136/bmj.n71

Table S3: Risk of Bias Assessment of Included Studies.

Study Name	Design	Randomization/ Confounding	Deviations/ Selection	Missing Data	Outcome Measurement	Selective Reporting	Overall Risk	Major Limitations Identified
**RCTs (ROB-2 Assessment):**								
Senapati et al. 2013	RCT	Low	Low	Some concerns	Low	Low	**Some concerns**	Slower recruitment, 16% lost to follow-up
Boccasanta et al. 2006	RCT	Low	Low	High	Low	Some concerns	**High**	Limited sample size, low study power
**Observational Studies (ROBINS-I Assessment):**								
Martucci et al. 2025	Retrospective	Serious	Serious	Low	Low	Low	**Serious**	Selection bias, multiple surgeons, short follow-up
Miura et al. 2024	Retrospective	Moderate	Serious	Moderate	Low	Low	**Serious**	Single institution, surgeon preference bias
Schabl et al. 2024	Cohort	Moderate	Moderate	Moderate	Low	Low	**Moderate**	Technique variations, limited follow-up
Jayalal et al. 2023	Prospective	Moderate	Low	Low	Moderate	Low	**Moderate**	Small sample, short follow-up, non-validated tools
Roy et al. 2023	Retrospective	Serious	Serious	Low	Low	Low	**Serious**	Non-randomized, small sample, reporting bias
Boccasanta et al. 2021	Retrospective	Low	Low	Low	Low	Low	**Low**	Large series, long follow-up, few drop-outs
Alwahid et al. 2019	Retrospective	Moderate	Serious	Low	Low	Low	**Serious**	No comparison group, retrospective design
Trompetto et al. 2019	Retrospective	Moderate	Low	Moderate	Moderate	Low	**Moderate**	Follow-up incomplete, non-validated scores
Pinheiro et al. 2016	Retrospective	Moderate	Low	Low	Low	Low	**Moderate**	Standard retrospective limitations
Elagili et al. 2015	Retrospective	Serious	Serious	Low	Low	Low	**Serious**	Non-randomized, small sample, low response
Kim et al. 2014	Retrospective	Moderate	Low	Low	Low	Low	**Moderate**	Standard retrospective design
Lee et al. 2014	Retrospective	Serious	Serious	Low	Moderate	Low	**Serious**	Referral bias, telephone assessment, small numbers
Ding et al. 2012	Retrospective	Moderate	Low	Low	Low	Low	**Moderate**	No functional outcomes, univariate analysis only
Ris et al. 2012	Prospective	Low	Low	Low	Low	Low	**Low**	Well-designed prospective study
Lee et al. 2011	Retrospective	Serious	Serious	Low	Low	Low	**Serious**	Skewed group sizes, short follow-up
Cirocco 2010	Retrospective	Moderate	Low	Low	Low	Low	**Moderate**	Standard retrospective limitations
de Oliveira Jr et al. 2010	Retrospective	Serious	Serious	Moderate	Low	Low	**Serious**	Small sample, recall bias, patients lost
Kim et al. 2010	Prospective	Low	Low	Low	Low	Low	**Low**	Well-conducted prospective study
Altomare et al. 2009	Retrospective	Moderate	Low	Low	Low	Low	**Moderate**	Standard retrospective design

Kimmins et al. 2001	Mixed	Moderate	Low	Low	Low	Low	**Moderate**	Mixed retrospective/prospective design
Takesue et al. 1999	Retrospective	Moderate	Low	Low	Low	Low	**Moderate**	Small series, standard limitations
Johansen et al. 1993	Prospective	Low	Low	Low	Low	Low	**Low**	Well-designed despite small size
Williams et al. 1992	Retrospective	Moderate	Low	Low	Low	Low	**Moderate**	Standard retrospective limitations

Abbreviations: *ROBINS-I, Risk Of Bias In Non-randomized Studies - of Interventions; ROB-2, Risk of Bias tool for randomized trials; RCT, randomized controlled trial.*

Risk of Bias Assessment (RoB 2.0) for Randomized Controlled Trials

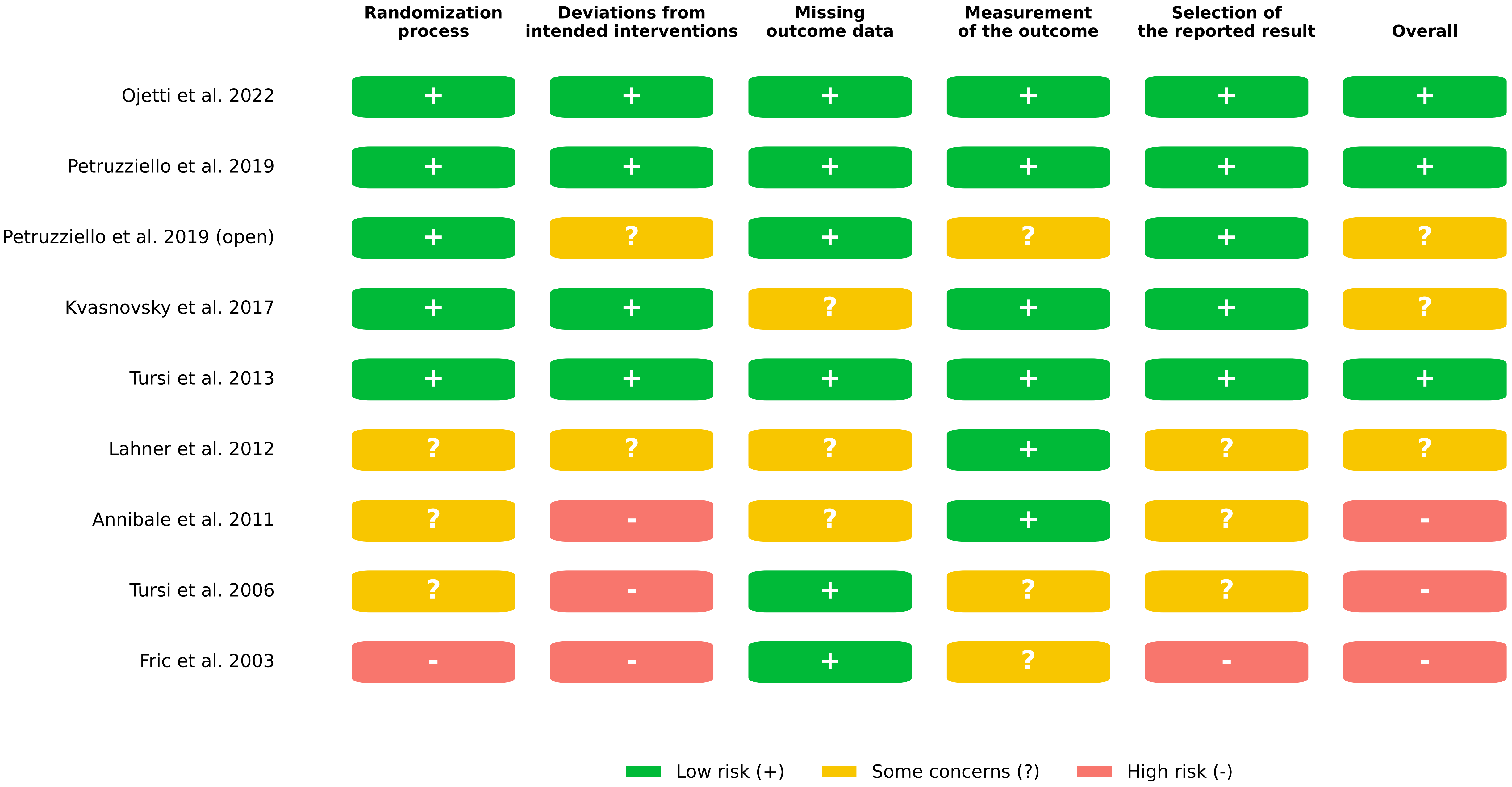

Risk of Bias Assessment (ROBINS-I) for Non-Randomized Studies

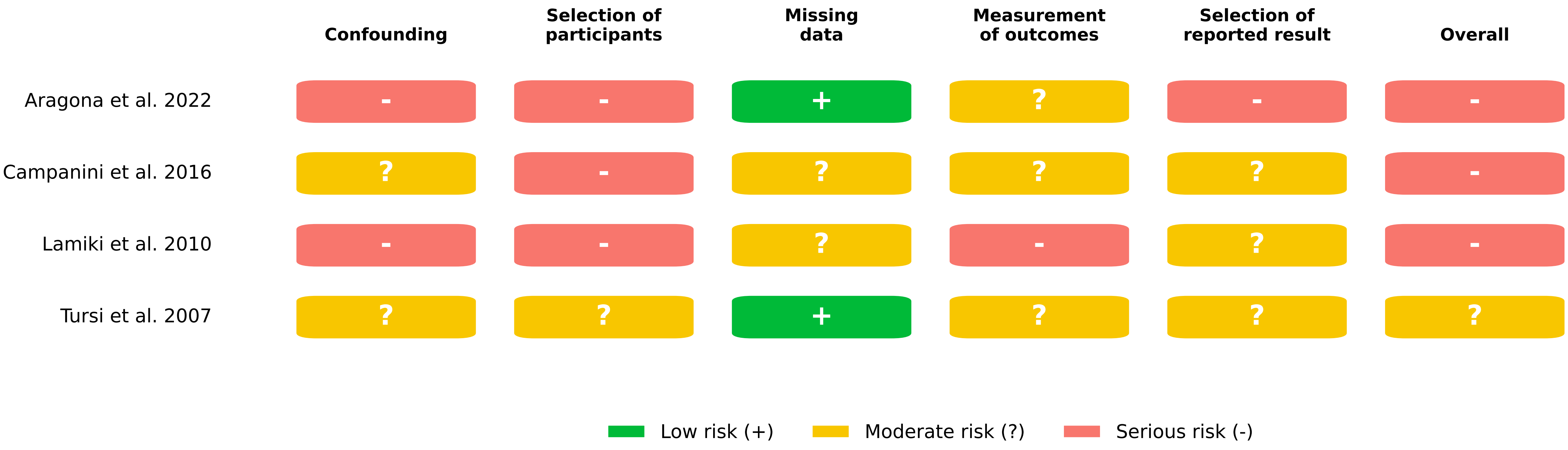

Table S4: GRADE Evidence Profile Outcomes.

Outcome	Studies (number)	Participants (number)	Starting Quality	Risk of Bias	Inconsistency	Indirectness	Imprecision	Publication Bias	Other Considerations	Final Quality
**Recurrence**	25	1,570	⊕⊕⊖⊖ LOW[1]	Serious (-1)[2]	Very serious (-2)[3]	Not serious (0)[4]	Not serious (0)[5]	Suspected (-1)[6]	Large effect (+1)[7]	⊕⊖⊖⊖ VERY LOW
**Major Complications**	19	1,279	⊕⊕⊖⊖ LOW[1]	Serious (-1)[2]	Serious (-1)[8]	Not serious (0)[4]	Not serious (0)[9]	Not suspected (0)[10]	None (0)	⊕⊖⊖⊖ VERY LOW
**Perioperative Mortality**	18	1,257	⊕⊕⊖⊖ LOW[1]	Serious (-1)[2]	Not serious (0)[11]	Not serious (0)[4]	Serious (-1)[12]	Not suspected (0)[10]	None (0)	⊕⊖⊖⊖ VERY LOW
**Recurrence: With Levatorplasty**	6	354	⊕⊕⊖⊖ LOW[1]	Serious (-1)[2]	Serious (-1)[13]	Not serious (0)[4]	Serious (-1)[14]	Not suspected (0)[10]	None (0)	⊕⊖⊖⊖ VERY LOW
**Recurrence: Without Levatorplasty**	4	78	⊕⊕⊖⊖ LOW[1]	Serious (-1)[2]	Serious (-1)[15]	Not serious (0)[4]	Very serious (-2)[16]	Not suspected (0)[10]	None (0)	⊕⊖⊖⊖ VERY LOW
**Recurrence: RCTs**	2	142	⊕⊕⊕⊖ MODERATE[17]	Not serious (0)[18]	Serious (-1)[19]	Not serious (0)[4]	Serious (-1)[20]	Not suspected (0)[10]	None (0)	⊕⊖⊖⊖ VERY LOW
**Recurrence: Observational**	23	1,428	⊕⊕⊖⊖ LOW[1]	Serious (-1)[2]	Very serious (-2)[21]	Not serious (0)[4]	Not serious (0)[5]	Suspected (-1)[6]	Large effect (+1)[7]	⊕⊖⊖⊖ VERY LOW

GRADE Criteria Explanations: *[1] **Starting Quality**: Observational studies start at LOW quality; RCTs start at HIGH quality [2] **Risk of Bias (Serious)**: Most studies retrospective with inherent selection bias, lack of blinding, incomplete follow-up [3] **Inconsistency (Very Serious)**: $I^2 = 76.3\%$, $p < 0.001$ indicating significant unexplained heterogeneity [4] **Indirectness (Not Serious)**: Population, intervention, and outcomes directly relevant to clinical question [5] **Imprecision (Not Serious)**: Large sample size (>1,500 patients) with narrow confidence intervals [6] **Publication Bias (Suspected)**: Asymmetric distribution of small studies, potential selective reporting [7] **Large Effect**: Absolute effect size >20% warrants upgrading by one level [8] **Inconsistency (Serious)**: $I^2 = 50.8\%$, $p = 0.006$ indicating moderate heterogeneity [9] **Imprecision (Not Serious)**: Adequate sample size with clinically meaningful precision [10] **Publication Bias (Not Suspected)**: Insufficient evidence of systematic bias in available studies [11] **Inconsistency (Not Serious)**: $I^2 = 0.0\%$, $p = 0.982$ indicating no heterogeneity [12] **Imprecision (Serious)**: Few events (n=10) leading to wide confidence intervals [13] **Inconsistency (Serious)**: $I^2 = 57.8\%$, $p = 0.036$ indicating moderate heterogeneity [14] **Imprecision (Serious)**: Moderate sample size with wide confidence intervals [15] **Inconsistency (Serious)**: $I^2 = 65.7\%$, $p = 0.032$ indicating significant heterogeneity [16] **Imprecision (Very Serious)**: Small sample size (n=78) with very wide confidence intervals [17] **Starting Quality (Moderate)**: RCTs start HIGH but downgraded for limitations [18] **Risk of Bias (Not Serious)**: RCTs with adequate randomization and methodology [19] **Inconsistency (Serious)**: $I^2 = 51.9\%$, $p = 0.149$ indicating moderate heterogeneity [20] **Imprecision (Serious)**: Small number of RCTs (n=2) with wide confidence intervals [21] **Inconsistency (Very Serious)**: $I^2 = 77.7\%$, $p < 0.001$ indicating significant heterogeneity. **Abbreviations:** CI, confidence interval; GRADE, Grading of Recommendations Assessment, Development and Evaluation; RCT, randomized controlled trial.*
